# Attenuation of quorum sensing system and virulence in *Vibrio cholerae* by phytomolecules

**DOI:** 10.3389/fmicb.2023.1133569

**Published:** 2023-03-30

**Authors:** Subhasree Saha, Shifu Aggarwal, Durg Vijai Singh

**Affiliations:** ^1^Regional Centre for Biotechnology, NCR Biotech Science Cluster, Faridabad, Haryana, India; ^2^Department of Infectious Disease Biology, Institute of Life Sciences, Bhubaneswar, Odisha, India; ^3^Department of Biotechnology, School of Earth, Biological and Environmental Sciences, Central University of South Bihar, Gaya, India

**Keywords:** quorum sensing, biofilm, biofilm matrix, inhibition, phytomolecules, therapy

## Abstract

The *Vibrio cholerae,* a gram-negative bacterium, is the causative agent of cholera. Quorum sensing is a cell-to-cell communication that leads to gene expression, accumulation of signaling molecules, biofilm formation, and production of virulence factors. The quorum sensing pathway in *V. cholerae* is regulated by *lux*O, and biofilm formation and other virulence factors are positively controlled by *aph*A and negatively by *hap*R. Hence, targeting the global regulator *lux*O would be a promising approach to modulate the QS to curtail *V. cholerae* pathogenesis. The present study investigated the modulating activity of quercetin and naringenin on biofilm formation and quorum-sensing regulated phenotypes in *V. cholerae*. Then after we determined the anti-quorum sensing capability of phytomolecules against the model organism *Chromobacterium violaceum*. Also, we performed flow cytometry for live/dead bacteria, MTT assay, CLSM, and growth curve analysis to determine their role as QS modulators rather than anti-bacterial. *V. cholerae* strains VC287 and N16961 formed thick biofilm. We observed a two-fold reduction in the expression of biofilm-associated genes comprising *gbpA, vpsA, rbmA*, and *mbaA* in the presence of phytomolecules indicating that phytomolecules modulate quorum sensing pathway rather than killing the bacteria. These phytomolecules were non-toxic and non-hemolytic and had anti-adhesion and anti-invasion properties. In addition, quercetin and naringenin were found to be highly effective compared to known quorum-sensing inhibitors terrein and furanone C-30. Thus, this study provides evidence that phytomolecules: quercetin and naringenin modulate the quorum-sensing pathway rather than killing the bacteria and can be used as an anti-quorum-sensing molecule for therapy against the pathogen.

## Introduction

*Vibrio cholerae*, a gram-negative, motile, and comma-shaped bacteria, is the causative agent of cholera. The disease is characterized by excessive watery stools, vomiting, and hypervolemic shock, and is life-threatening if left untreated (Elimian et al., 2019). This organism is a natural inhabitant of the aquatic environment from which it enters the human host *via* the fecal-oral route or by consuming contaminated water. There were 1.3–4.0 million cases of cholera and 21,000 to 143,000 deaths worldwide due to *V. cholerae* infection every year^1^.

Over the years, the emergence of extensive drug resistance (XDR) and multidrug-resistant (MDR) *V. cholerae* strains possess a public health concern owing to their association with treatment failures (Qadri et al., 2017). Thus, there is a need for developing novel strategies to combat *V. cholerae* infection. One of the main reasons for bacterial resistance is the formation of biofilms. The *V. cholerae* biofilm allows it to survive under stress conditions within the host. It prefers a biofilm mode of growth in the aquatic environment and intestinal tract of human beings (Yildiz and Visick, 2009). It has been reported that the major component of *V. cholerae* biofilm is Vibrio polysaccharide that is synthesized by enzymes encoded in two *vps* (Vibrio polysaccharide synthesis) operons (*vps-I* and *vps-II*) (Fong and Yildiz, 2007). The genes *rbmA, rbmB,* and *rbmC* located in the intergenic region of the operons, and *bap1* located downstream of *vps-II* are involved in the production of matrix proteins and maintenance of biofilm structure (Pederson et al., 2018).

The biofilm-like aggregates formed by *V. cholerae* during host colonization are associated with virulence and are essential in pathogenesis and disease transmission (Silva and Benitez, 2016). The biofilm aggregates that aid in cellular attachment is critical virulence factors. The other factor for *V. cholerae* survival is motility which helps the bacteria to adhere to the host for infection and colonization (Ali et al., 2015). It has been reported that non-motile mutant strains of *V. cholerae* have reduced virulence which correlates with reduced motility and weakened adherence capability of the bacterial cells to the host (Guentzel and Berry, 1975).

It has been demonstrated that *V. cholerae* uses quorum sensing to regulate biofilm formation and virulence mechanisms (Teschler et al., 2015). Thus, manipulating the QS system of *V. cholerae* using target-specific inhibitors could be a promising anti-virulence therapeutic approach (Górski et al., 2016). In *V. cholerae*, the quorum sensing system is initiated by two sets of autoinducers in a parallel namely cholera autoinducer 1 (CAI-1), autoinducer 2 (AI-2), and their cognate inner membrane receptors cqsS and luxP/Q (Rutherford et al., 2011). The virulence factors of *V. cholerae* are expressed at a low cell density (LCD) state. However, at high cell density (HCD) the expression of virulence factors is down-regulated to enhance the production of the enzyme protease that detaches vibrios from the human intestine (Hammer and Bassler, 2003; Jung et al., 2016). The receptors CqsS and LuxP/Q possess both kinase and phosphatase activities and transfer information to a transcriptional response regulator LuxO (Zhu et al., 2002; Papenfort and Bassler, 2016). The regulator LuxO, acts as a genetic switch between low and high concentrations of their respective signals (Sarveswari and Solomon, 2019).

In recent times, the overuse of conventional antibiotics has led to the emergence of multidrug-resistant bacteria (Eibach et al., 2016). Compared to conventional antibiotics, plant-derived molecules, especially phytochemicals that are isolated from traditionally used medicinal plants have gained importance due to their anti-infective potential against infectious diseases (Akkoç et al., 2015). The bacterial QS system has been evaluated as the new target for developing anti-infective therapies since blocking of QS would weaken bacterial virulence, thus making them susceptible to treatment and easy clearance by host defense mechanisms (Husain et al., 2017). Hence, the detection of phytomolecules interfering with QS and virulence may act as promising anti-infective compounds (Abirami et al., 2021).

In this study, we investigated the biofilm-forming ability of *V. cholerae* strains and the ability of phytomolecules to inhibit biofilm formation in *V. cholerae*. Also, the anti-quorum sensing potential of two phytomolecules, quercetin and naringenin against *V. cholerae* strains was studied.

## Materials and methods

### Bacterial strains and phytomolecules

The strains used in this study were the *V. cholerae* O1 VC287 obtained from a diarrheal patient from Thiruvananthapuram, India, and El Tor strain N16961. All the strains were characterized earlier using standard biochemical and genetic methods and maintained in Luria-Bertani broth (Difco) supplemented with 30% glycerol at –80°C or Luria-Bertani agar stab culture at room temperature. The detailed characteristic of strains is given in Table 1. The phytomolecules naringenin (N5893-25G) and quercetin (Q4951-100G) were acquired from Sigma.

**Table 1:** Biochemical and genetic characteristic of *V. cholerae* strains used in the study.

Strains	Serotype	Biotype	Year of isolation	Presence of genes encoding for				
				*ctxB*	*rstR*	*hapR*	*hlyA*	*tcpA*
*V. cholerae* N16961	O1	El Tor	1972	+	+	+	+	+
*V. cholerae* VC287	O1	El Tor	2002	+	+	+	+	+

### Semi-quantitative estimation for biofilm formation

The *V. cholerae* biofilm was quantified by the method described by O'Toole (2011) with certain modifications. Briefly, the overnight grown culture of *V. cholerae* strains was diluted 100 times and inoculated in 200 μl of fresh LB broth contained in 96-well flat-bottom polystyrene plates (COSTAR; Sigma). The cells were grown for 24 h, at 37°C under static conditions. The supernatant was removed, and the biofilm was washed three times with PBS. Adherent cells were then stained with 0.1% crystal violet, washed thoroughly with water, and dissolved in 33% glacial acetic acid. The biofilm formation was measured at OD_570_ nm in a Multimode reader (Nivo). Strains were considered biofilm positive if the strain had an OD ≥1.0. The experiment was performed in triplicates, and the data showing values *p* > 0.05 were omitted. The biofilm morphology was determined using scanning electron microscopy (SEM) and confocal laser scanning microscopy (CLSM).

### Scanning electron microscopy

We performed the scanning electron microscopy following the method of Townsley et al. (2016) with certain modifications. Briefly, the overnight cultures were diluted, and the treated and untreated cultures of *V. cholerae* strains were added in a 35 mm petri dish (Corning, Costar) containing 18 mm coverslip at an OD_570_ of 0.5 in LB and incubated at 37°C for 24 h. The bacterial cells were fixed with 2.5% glutaraldehyde for 2 h at room temperature. After fixation, the coverslips were washed thrice with 1X PBS for 5 min. The bacterial cells were subjected to a series of graded ethanol dehydration (30, 50, 70, 80, 95, and 100%) for 5 min each, respectively, followed by critical point drying using Quorum K850. The coverslips were coated with gold–palladium alloy acquired in a Denton DV503 vacuum apparatus (Denton Ltd. Cherry Hill, NJ, United States). The samples were examined in a Zeiss scanning electron microscope (Advanced Metals Research Corp. Bedford, MA, United States) at an accelerating voltage of 15 kV. The Zeiss SEM camera was used to capture photographs.

### Confocal laser scanning microscopy

The phytomolecules naringenin and quercetin treated and untreated *V. cholerae* strains were grown for 24 h at OD_570_ of 0.5 on a six-well plate containing 18 mm coverslip and incubated at 37°C. The coverslips were washed thrice in 1X PBS and stained with acridine orange (Sigma, 0.02%) for 30 min in the dark at room temperature. The cells were fixed with 4% paraformaldehyde, incubated for 30 min in the dark, and collected images by CLSM STED (Leica TCS-SP8). The fluorescence of acridine orange was detected by excitation at 488 nm and emission with a 500–530 nm bandpass filter. The z-stacks at one-um intervals were collected and exported as .tif files from Leica LAS AF (version 1.8.2) software and imported into the ImageJ (version 1.41) program (USNIH, Bethesda, MD, United States). We used the PHILIP program to analyze the randomly selected five different fields for imaging and quantification of the mean thickness of biofilm.

### Antibiofilm activity of the phytomolecules against preformed biofilms

We determined the effect of naringenin, quercetin, pyrogallol, optochin, magnolol, herperidine (Sigma) on a performed biofilm by *V. cholerae* El Tor N16961, and VC287 in a 24-well plate (Corning, Costar) for 24 h (Lee et al., 2013). The plates were incubated further for another 24 h at 37°C under static conditions, washed once with 1X PBS, and then stained with 0.1% crystal violet for 30 min at room temperature. The bound dye was dissolved in 95% ethanol, and the absorbance was recorded at 570 nm (Nivo Multimode reader). The reduction percentage of growth or biofilm was shown by using the following formula (Sivaranjani et al., 2016).

Reduction percentage=[(ControlOD−treatedOD)/controlOD]×100

Based on the effect of the inhibitors on the biofilm, the effect of quercetin and naringenin on the quorum sensing of *V. cholerae* strains VC287 and N16961 was further determined.

### Violacein inhibition assay

Inhibition of quorum sensing by quercetin and naringenin was performed with agar well diffusion assay using *Chromobacterium violaceum* CV026 as an indicator strain (Mclean et al., 2004). Briefly, overnight culture of CV026 (0.5% v/v) was inoculated in Luria-Bertani soft agar (0.5% v/v) which was laid on LB agar (17% v/v) and incubated for 24 h at 37⁰C. Wells of 7 mm diameter were made on the solidified LB soft agar and were loaded with different concentrations of quercetin and naringenin (50, 75, 100, 125, 150 μg/ml). The plates were incubated for 24 h at 37⁰C. The inhibition of violacein synthesis was detected by a turbid or creamy ring around the well against the violet color of the *Chromobacterium violaceum* culture. Depending on the results from initial dose dependent screening, 50 μg/ml which is half-MBIC of the phytomolecules was used for further experiments.

### Quantitative estimation of violacein

The quantitative analysis of QS-controlled production of violacein by *C. violaceum* was quantified spectrophotometrically after treatment with quercetin and naringenin at concentrations 50, 75, 100, 125, and 150 μg/ml (Blosser and Gray, 2000). Briefly, 1 ml of 16–18 h bacterial culture (OD_600nm_ = 0.1) of *C. violaceum* (CV026) was suspended in 20 ml of LB broth containing different concentrations of inhibitors and incubated at 37°C for 24 h. The tubes were vortexed vigorously for the extraction of violacein pigment. The bacterial cell debris was removed by centrifugation and the absorbance of soluble violacein was determined using a microtiter plate reader (Nivo, Perkin Elmer). The percentage inhibition of violacein production in the presence of plant extracts was measured as, [(OD of control – OD of treated)/OD of control] × 100 (Husain et al., 2017). After the initial dosage screening the 50 μg/ml which is half of minimum biofilm inhibitory concentration (MBIC) were used for further experiments.

### Flow cytometry analysis

The bacterial cell viability was determined using the propidium iodide staining method as described by Singh and Barnard (2021) with some modifications. Briefly, *V. cholerae* strains VC287 and N16961 were grown in the presence of quercetin and naringenin (50 μg/ml) at 37°C for 24 h. The overnight grown culture was diluted 500 times with LB broth to an OD_600nm_ = 1. Cells were incubated in LB for 15 min at room temperature with 15 μg/ml of propidium iodide (Thermo Fischer Scientific, United Kingdom). The cells were washed with PBS 2 times and resuspended in 1X PBS. The proportion of PI-positive events was distinguished within SSC/FSC selected bacterial population. The data was acquired using FACS Calibur flow cytometry (Becton Dickinson) and analyzed by Cell Quest Pro software (Giacomucci et al., 2019). The percentage of live cells was calculated by subtracting the percentage of dead cells from the total gated population for the strains VC287 and N16961.

### Biofilm metabolic activity assay

The metabolic activity of biofilm was determined by the reduction of MTT assay according to the protocol of Denizot and Lang (1986) with some modifications. Overnight grown cultures of *V. cholerae* strains were diluted 100 times and inoculated in 200 μl of fresh LB broth contained in 96-well flat-bottom polystyrene plates in the presence or absence of quercetin and naringenin Half-MBIC concentrations (COSTAR; Sigma). After 24 h of incubation at 37°C under static conditions, the culture was removed and washed thrice with water. The MTT salt (Sigma-Aldrich, United States) was dissolved in phosphate-buffered saline (PBS) to give a final concentration of 5 mg ml^−1^. The culture medium was carefully removed, and the wells were washed five times with 0.85% (w/v) NaCl solution and air-dried. One hundred microliters of MTT solution were pipetted into each well and incubated for 3 h at 37°C. The insoluble purple formazan obtained by enzymatic hydrolysis of MTT by the dehydrogenase enzyme found in living cells was further dissolved in 100 μl of dimethyl sulphoxide (DMSO; Sigma-Aldrich, United States). The absorbance was then measured at 570 nm using a microplate reader (Nivo, Multimode).

### RNA isolation and qRT-PCR profiling for gene expression

The expression of biofilm-associated genes *gbpA*, *vpsA*, *rbmA*, and *mbaA* and quorum sensing genes *luxO*, *cqsA*, *cqsS*, *hapA*, *hapR*, *aphA*, *tcpA* was determined by qRT-PCR. The aliquots of 12 h grown culture in LB diluted to an absorbance of 0.05 OD at 600 nm were seeded in a 6-well microtiter plate (Corning, Costar) with and without inhibitors; quercetin (50 μg/ml), naringenin (50 μg/ml), and pyrogallol (50 μg/ml) and incubated at 37°C for 24 h under static conditions. The adherent cells were scraped using PBS and centrifuged at 18,000 *g* for 5 min at 4°C. RNA was isolated from the pellet using a Trizol reagent (Ambion), as per the manufacturer’s instructions. The cDNA was synthesized using 500 ng RNA and a cDNA synthesis kit (GeneX). The cDNA was diluted 30 times using nuclease-free water (1:30) and 1 μl of cDNA was used as a template in RT-PCR (Panda and Singh, 2018). The cycling conditions were as follows: initial denaturation at 95°C for 5 min, followed by 35 cycles of 95°C for 15 s and 53°C to 60°C for 30 s, and final extension at 72°C for 2 min. The fold difference was determined using the 2^–ΔΔCT^ method (Pfaffl, 2001). Amplification, data acquisition, and relative expression analysis are carried out in a Quant 6 system (Applied Biosystems).

#### Swimming motility assay

Motility assay was performed by modifying a method described previously (Vattem et al., 2007). Briefly, motility agar plates were made from 1% (w/w) glucose, 0.3% (w/v) Bacto agar, 0.5% (w/v) Bacto peptone, and 0.2% (w/v) yeast extract. The phytomolecules, quercetin, and naringenin were added at a concentration of 50 μg/ml on the top of the 0.3% LB agar plates. The plates were point inoculated with *V. cholerae* overnight culture (OD_600nm_ = 0.1) and incubated at 37°C for 16 h. The diameter of *V. cholerae* motility indicates the anti-virulence properties of drugs. The assay was done in technical duplicates and at least biological triplicates.

#### Adhesion assay

HeLa cells were grown to confluence in DMEM medium containing 10% FBS and 1% antibiotics. Cells were grown in 24 well plates and washed three times with sterile PBS and DMEM medium. In parallel, *V. cholerae* was grown to the mid-exponential phase in the presence and absence of quercetin and naringenin at the 50 μg/ml. The cells were pelleted and resuspended in DMEM medium containing 10% FBS without antibiotics. The culture was then added to the HeLa cells monolayer at a multiplicity of infection (MOI) of 50 and incubated for 90 min at 37°C in a 5% CO_2_ atmosphere, for bacterial adherence to the cell monolayer. The non-adherent bacteria were removed by washing the cell lines repeatedly with PBS and DMEM containing 10% FBS. After repeated washing, the adherent cells were then detached using 0.1% Triton X-100 in PBS. The bacterial CFU was determined by serial plating. All experiments were performed in triplicates (Hema et al., 2017).

#### Invasion assay

Invasion experiments were performed using *V. cholerae* strains grown in presence of quercetin and naringenin at 50 μg/ml concentration. Briefly, overnight grown cultures in LB medium were diluted 1:100 in fresh broth containing quercetin and naringenin at 50 μg/ml, and incubated for 16 h at 37°C. Bacterial cells were harvested by centrifugation; the pellets were resuspended in PBS and re-centrifuged. The final pellets were resuspended in HeLa cells in DMEM medium containing 10% FBS and 1% antibiotics at a multiplicity of infection (MOI) of 50 and incubated for 1 h at 37°C. To determine viable intracellular bacteria, infected cells were washed 3 times with PBS and covered with 1 ml DMEM containing gentamicin (100 μg/ml). After 3 h at 37°C in 5% CO_2_, monolayers were washed and the cell lines were lysed with Triton X-100, then viable internalized bacteria (CFU/ml) were counted by plating the cells on LB agar. Each assay represented the average of triplicate wells. All experiments were performed thrice (Facinelli et al., 1998).

#### Hemolysis assay

Hemolytic activity was measured as described previously (Smith-Palmer et al., 2004). Briefly, 1 ml of culture supernatant of strains grown overnight in presence of quercetin and naringenin at 25, 50, 75, and 100 μg/ml was incubated with 485 μl PBS, to which sheep erythrocytes were added to achieve a final concentration of 1%; 100% hemolysis was obtained with 1% erythrocytes in water. Samples were incubated at 37°C for 30 min, centrifuged (1,000 × *g*) for 5 min, and read by a spectrophotometer at a wavelength of 690 nm. Results were expressed as a percentage of hemolysis for the control strain, grown without the inhibitors. The criteria for toxicity were non-toxic (0–9%), slightly toxic (10–49%), toxic (50–89%), and highly toxic (90–100%) as previously described by Salni et al. (2002).

#### Cytotoxicity assay

The toxicity of quercetin and naringenin was determined at different concentrations by MTT assay using HeLa cells according to Saha et al. (2013). HeLa cell line was cultured in DMEM medium (Dulbecco’s modified Eagle’s medium) (Sigma-Aldrich) with 10% fetal bovine serum (FBS) and streptomycin–penicillin (100 U/ml). The culture was maintained in a 5% CO_2_ incubator at 37°C. The cell viability was evaluated using the MTT assay (Sigma-Aldrich) according to the manufacturer’s protocol. Briefly, the cell line was seeded into a 96-well microtiter plate (Nunc) at a cell density of 1 × 10^4^ cells/well and allowed to attach for 24 h at 37°C. The culture medium was replaced with 200 μl of fresh DMEM medium containing different concentrations of quercetin and naringenin. The cells were then incubated with the appropriate concentration of inhibitors for 3 h with FBS used as a negative control. The absorbance was measured at 540 nm, and the mean absorbance value of the untreated cells was considered 100% cellular viability. The viable cells after treatment with inhibitors were expressed as a percentage of the untreated control.

### Statistical analysis

All experiments were performed in triplicates and the standard deviation from the mean was calculated. The effect of the inhibitors was analyzed using One-way ANOVA by GraphPad Prism Version 8 software. Values were considered significantly different at *p* < 0.05.

## Results

### Biofilm formation

**Figure 1:**
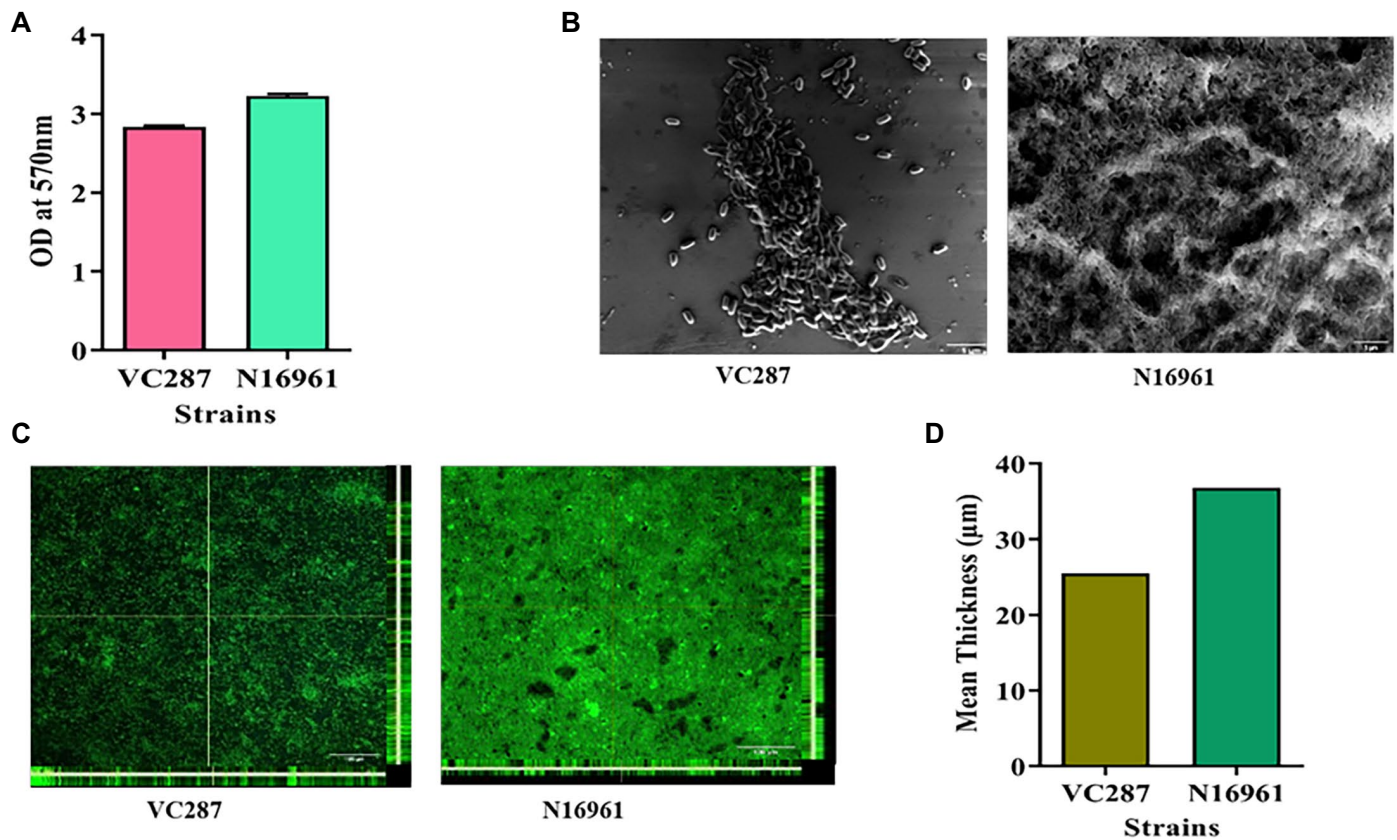
Biofilm formation in *Vibrio cholerae* strains. **(A)** Crystal violet staining of 24-h grown culture of *V. cholerae* strains VC287 and N16961. **(B)** Scanning electron microscopy of the 24-h grown culture of VC287 and N16961. **(C)** Orthogonal views obtained by CLSM after 24-h grown culture of VC287 and N16961. The *z*-stacks were acquired for each chamber by CLSM with a Leica TCS SP5 confocal scanning system (Leica Microsystem, Mannheim, Germany) using 63× oil objective lens at 68x. **(D)** CLSM analysis represented the mean thickness of biofilm formed by *V. cholerae* strains.

The *V. cholerae* strains VC287 and N16961 produced thick biofilm with an OD_570_ value ranging from 2.0–3.0 (Figure 1A). These findings were confirmed by Scanning Electron Microscopy and Confocal Laser Scanning Microscopy (Figures 1B,C). The biofilm-forming capacity of these isolates was further quantified by calculating the mean thickness of biofilm by CLSM using the Image J software (Figure 1D).

### Screening of the anti-biofilm properties of phytomolecules

**Figure 2:**
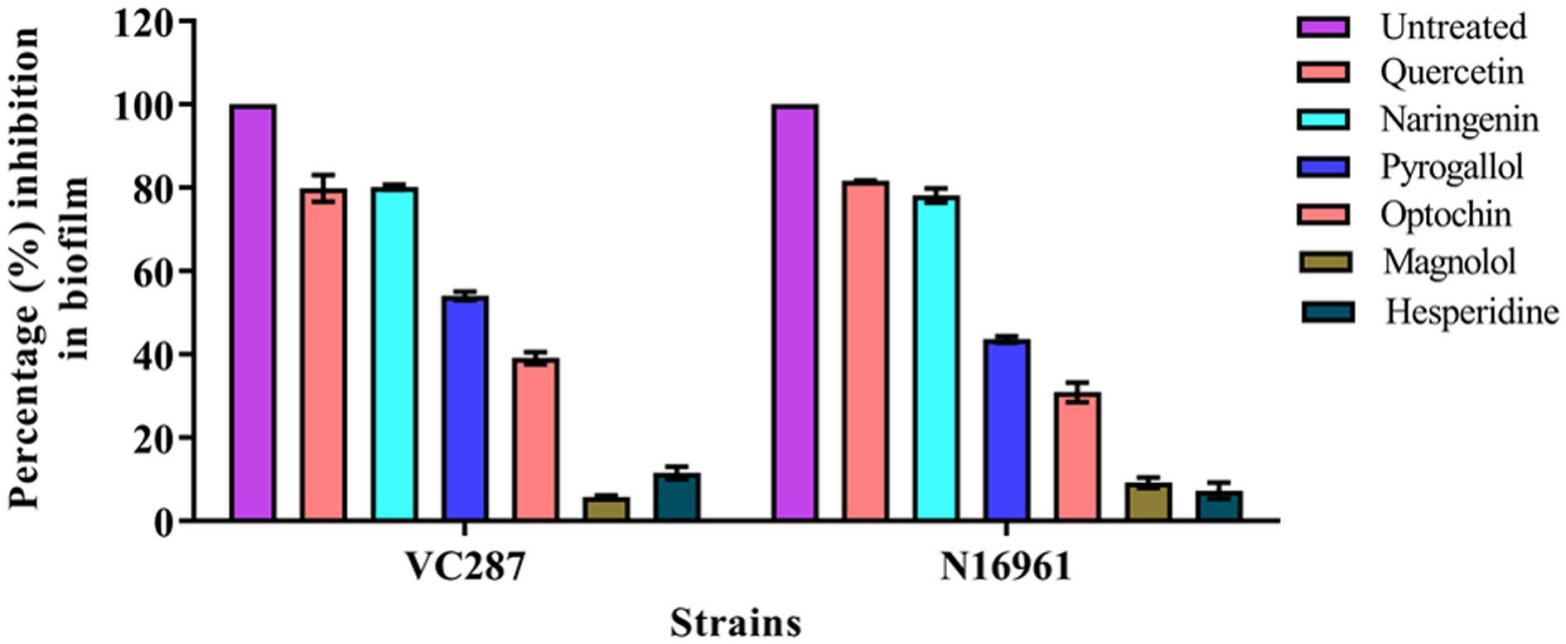
Biofilm inhibition by phytomolecules. Results showed the effect of quercetin, naringenin, pyrogallol, optochin, magnolol, and hesperidine on biofilm formation by *V. cholerae* strains VC287 and N16961.

A crystal violet assay was performed to determine the effects of phytomolecules on biofilm formation. *V. cholerae* strains VC287 and N16961 when cultured with quercetin and naringenin at concentration 50 μg/ml, 70–80% reduction in biofilm formation was observed. *V. cholerae* strain N16961 showed a 20–30% reduction in biofilm formation when treated with optochin whileVC287 showed a 60% reduction in biofilm formation. However, magnolol and hesperidin did not show any effect on biofilm formation (Figure 2). Therefore, two phytomolecules, quercetin, and naringenin were selected as a candidate for further study.

### Screening of inhibitors for their ability to inhibit violacein production

**Figure 3:**
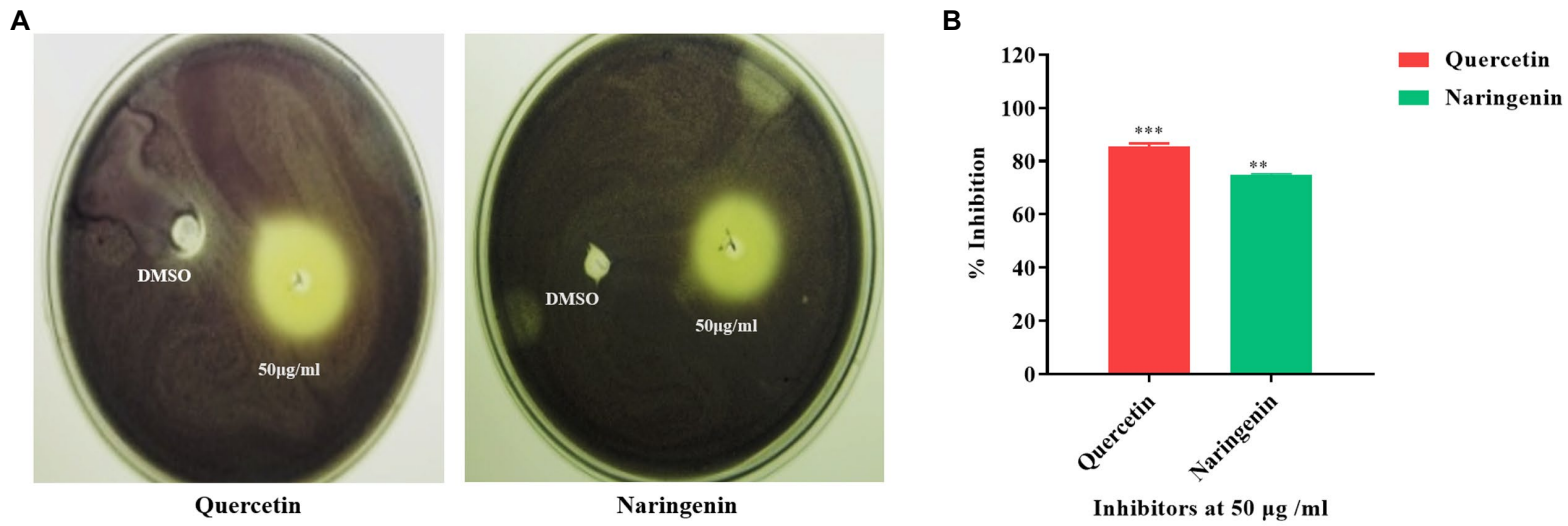
Quorum sensing inhibition obtained with *C. violaceum* CV026 by quercetin and naringenin. **(A)** The zone of inhibition obtained at 50 μg/ml concentration of quercetin and naringenin with DMSO as control. **(B)** Quantitative estimation of violacein production (%) with and without quercetin and naringenin. **, *** denotes level of significance.

The inhibitors were tested for their ability to inhibit the QS-dependent production of violacein in *C. violaceum* CV026. Cells that were affected by the inhibitors diffused from the well to the surrounding medium and thus failed to produce violacein. While cells that grew without the influence of inhibitors were violet in color because of QS-guided production of violacein. We found quercetin and naringenin at 50 μg/ml concentration produced halo zones of 35 mm and 33 mm diameter indicating their anti-QS activity (Figure 3A). The quantitative estimation of violacein in culture supernatant showed the maximum inhibition of *C. violaceum* with quercetin was 80 ± 3.15% and for naringenin, it was 75 ± 4.35% (Figure 3B). These findings indicate that quercetin and naringenin can be used as QS inhibitors in the study.

### Growth curve analysis

**Figure 4:**
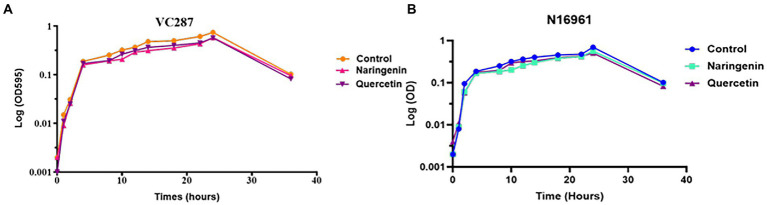
Growth curve analysis of *Vibrio cholerae* strains. **(A)** VC287 and **(B)** N16961 in the absence or presence of quercetin and naringenin at 50 μg/ml concentration.

The growth of *V. cholerae* strains VC287 and N16961 was evaluated at 50 μg/ml of quercetin and naringenin (Figure 4). In VC287, a slight difference was first observed between untreated and treatment groups before 20 h. Although there was some distinction between inhibitors and the untreated group after 24 h but it was not significant. In case of strain N16961, no significant difference was found in between the treated and untreated group throughout 36 h. The lack of variation in growth pattern and constrained growth compared to untreated indicates its ability to attenuate the virulence properties of *V. cholerae* rather than killing of the pathogen.

### Dose-dependent inhibition of biofilm formation

The preformed biofilm was treated with quercetin and naringenin at concentrations from 25 μg/ml to 125 μg/ml. The strains N16961 and VC287 showed 15–25% reduction in biofilm when treated with a concentration of 25 μg/ml of quercetin and naringenin. These strains when treated with quercetin and naringenin at 50 μg/ml concentration showed 60–70% reduction in biofilm formation. At 75 μg/ml to 125 μg/ml concentration, quercetin and naringenin showed 80–100% reduction in biofilm formation (Supplementary Figures S1A,B). Thus, we used 50 μg/ml concentration of quercetin and naringenin for our further study.

Further, the time point inhibition studies with quercetin and naringenin at 50 μg/ml were performed at 0, 8, 12, 16, 20, and 24 h. There was no significant effect of quercetin and naringenin on biofilm formation at the initial time point of 0–8 h. However, these strains showed a 30–40% reduction in biofilm when treated with quercetin and naringenin at 12 and 16 h. At 20–24 h quercetin and naringenin-treated cells showed a 50% reduction in biofilm (Supplementary Figures S1C,D). The data from this study was further confirmed by SEM and CLSM which showed a reduction of biofilm formation in both the strains (Supplementary Figures S2A–C).

### Bacterial cell viability assays

**Figure 5:**
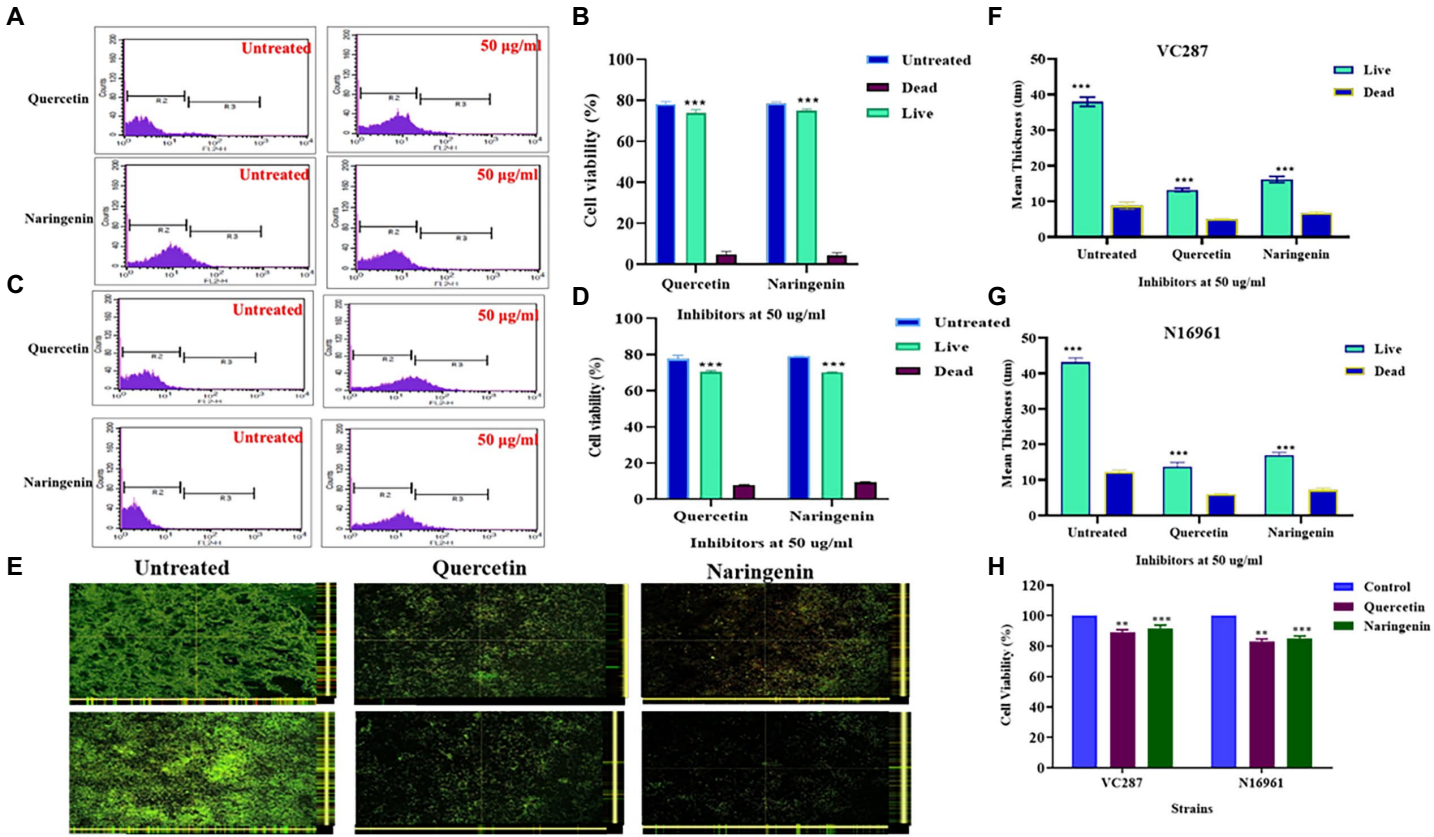
Cell viability analysis of *V. cholerae* strains VC287 and N16961 after treatment with phytomolecules. **(A,B)** Flow cytometry viability analysis of *V. cholerae* strain VC287. **(C,D)** Flow cytometry viability analysis of *V. cholerae* strain N16961. Estimation of the frequency of live and dead cells after treatment with quercetin and naringenin at 50 μg/ml concentration using FACS. **(E)** Confocal microscopy images of Live/Dead staining of biofilms formed by *V. cholerae* strains VC287 and N16961 in the presence or absence of quercetin and naringenin at 24 h. **(F)** The live bacteria present in biofilm is shown in Sea Green color and scattered dead bacteria were shown in blue color. **(G)** Live/dead staining is represented as the mean thickness of live and dead bacteria present in the biofilm with and without inhibitors. **(H)** Viability of HeLa cells determined by MTT assay after exposure to quercetin and naringenin at 50 μg/ml for 24 h. **, *** denotes level of significance.

Propidium iodide staining and FACS analysis of untreated and treated cells in biofilm showed that quercetin and naringenin had less number of dead cells. Figures 5A–D showed 75–80% of cells were viable after treatment with quercetin and naringenin thus validating their anti-quorum sensing properties. Live dead staining of untreated and treated biofilm with inhibitors showed a reduction in the overall biofilm formation in the stains VC287 and N16961 (Figure 5E), however less number of dead cells was observed (Figures 5F,G). Similarly, quercetin and naringenin-treated *V. cholerae* strain VC287 and N16961 showed significant metabolic activity in the range of 85–90% compared to untreated cells (Figure 5H). These observations suggest that quercetin and naringenin had quorum-sensing inhibitor activity.

### Expression analysis of biofilm and quorum-associated genes

**Figure 6:**
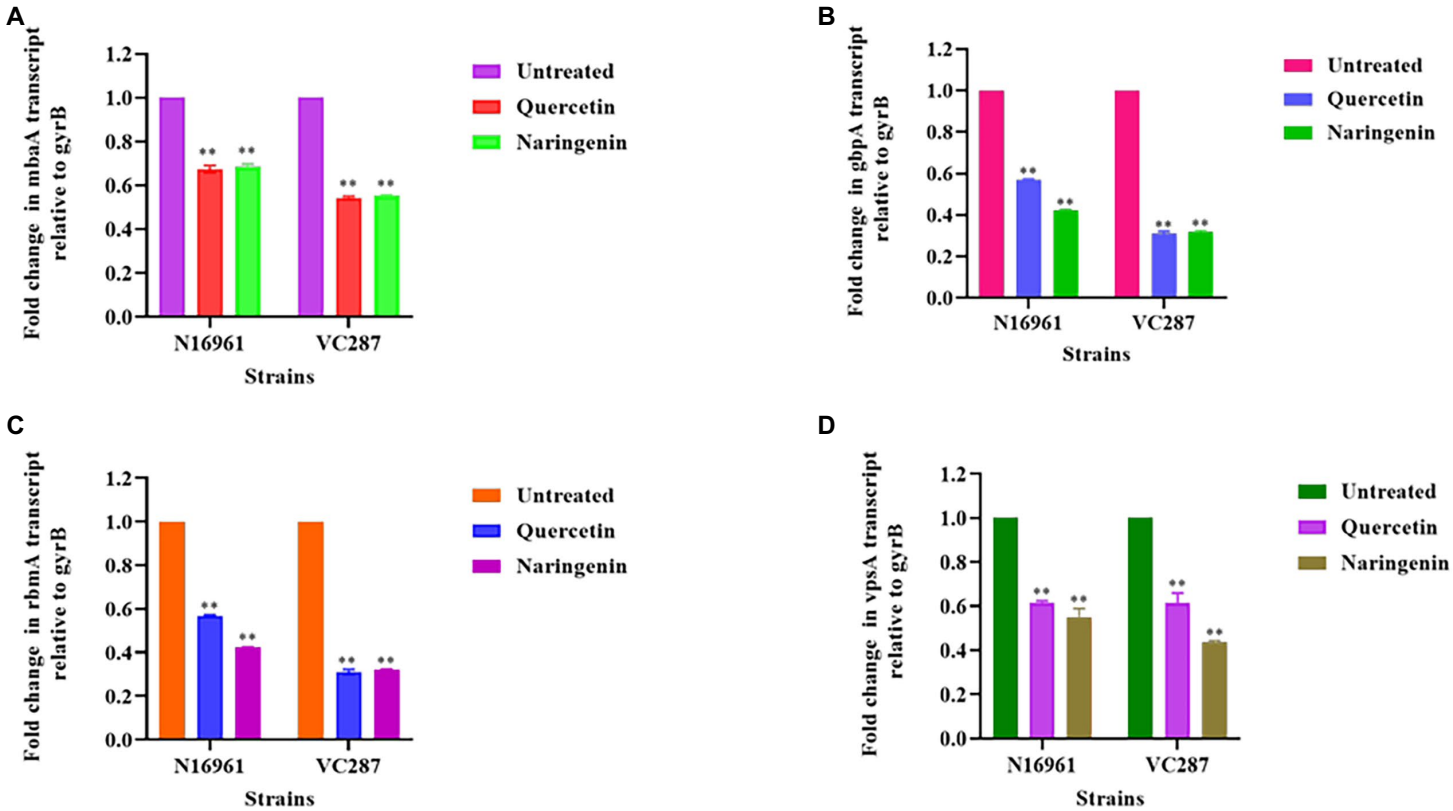
Relative expression of biofilm associated genes measured by real-time qPCR. **(A)** *mbaA*; **(B)** *gbpA*; **(C)** *rbmA*; **(D)** *vpsA*. The fold change in biofilm gene expression was normalized to the level of *gyrB* gene expression and then quantitated the relative gene expression of control normalized to 1 using the comparative Ct method. Results were the means and SD obtained from three experiments. *p < 0.05 and **p < 0.01 indicate statistically significant differences between treated and control.

**Figure 7:**
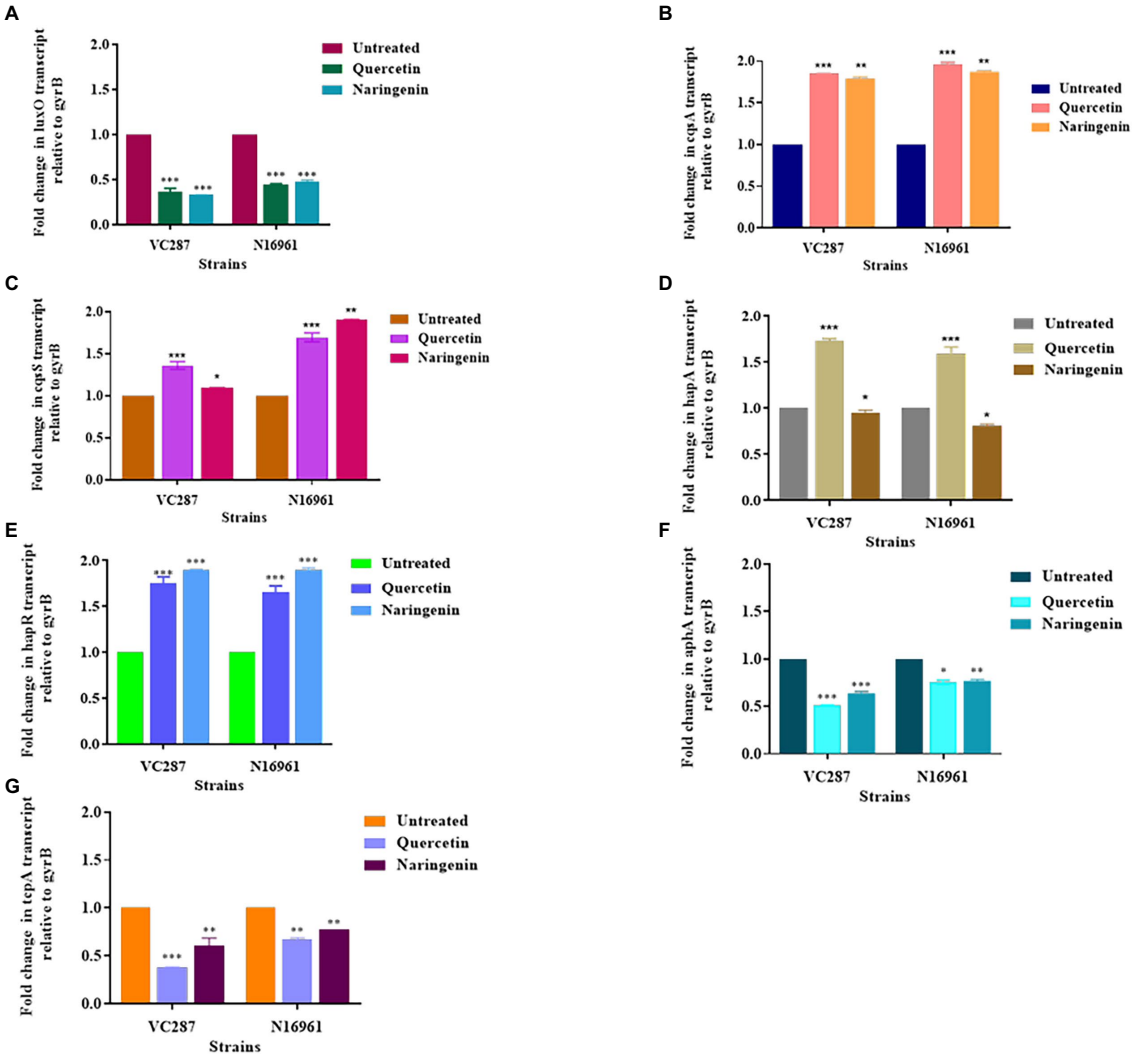
Relative expression of quorum-sensing genes measured by real-time qPCR. **(A)** *luxO*; **(B)** *cqsA*; **(C)** *cqsS*; **(D)** *hapA*; **(E)** *hapR*; **(F)** *aphA*; **(G)** *tcpA*. The fold change in QS gene expression was normalized to the level of *gyrB* gene expression and then quantitative relative gene expression of control normalized to 1 using the comparative Ct method. Results represent the means and SD obtained from three experiments. *p < 0.05 and **p < 0.01 indicate statistically significant differences between treated and control. **, *** denotes level of significance.

The expression study of biofilm-associated genes *gbpA, vpsA, rbmA*, and *mbaA* in the presence of quercetin and naringenin at 50 μg/ml concentration showed that the inhibitors affected the biofilm matrix architecture, maintenance, bacterial adhesion, and host colonization. *V. cholerae* strains N16961 and VC287, when treated with quercetin and naringenin, showed two-fold downregulation in the expression of the *gbp*A, *vps*A, and *mbaA*. However, the *rbm*A expression was reduced by two-fold in both strains N16961 and VC287 after treatment with quercetin and naringenin (Figure 6). The relative expression of quorum sensing associated genes was determined to understand the effect of inhibitors on the *luxO* gene. The quercetin and naringenin showed a two-fold increase in the expression of *cqsA, cqsS, hap*R, and *hapA* genes but a decrease in l*uxO, tcpA* and *aphA* genes (Figure 7). These observations led to a hypothesis that QS modulators inducing accumulation of CAI-1 autoinducers, triggered a phosphorelay cascade in the LCD state to mimic the HCD state resulting in the reduction of virulence factor, biofilm formation, and early detachment and dissemination of *V. cholerae* cells, however, the exact mechanism of action of the phytomolecules will require further investigations.

### Anti-virulence assays

**Figure 8:**
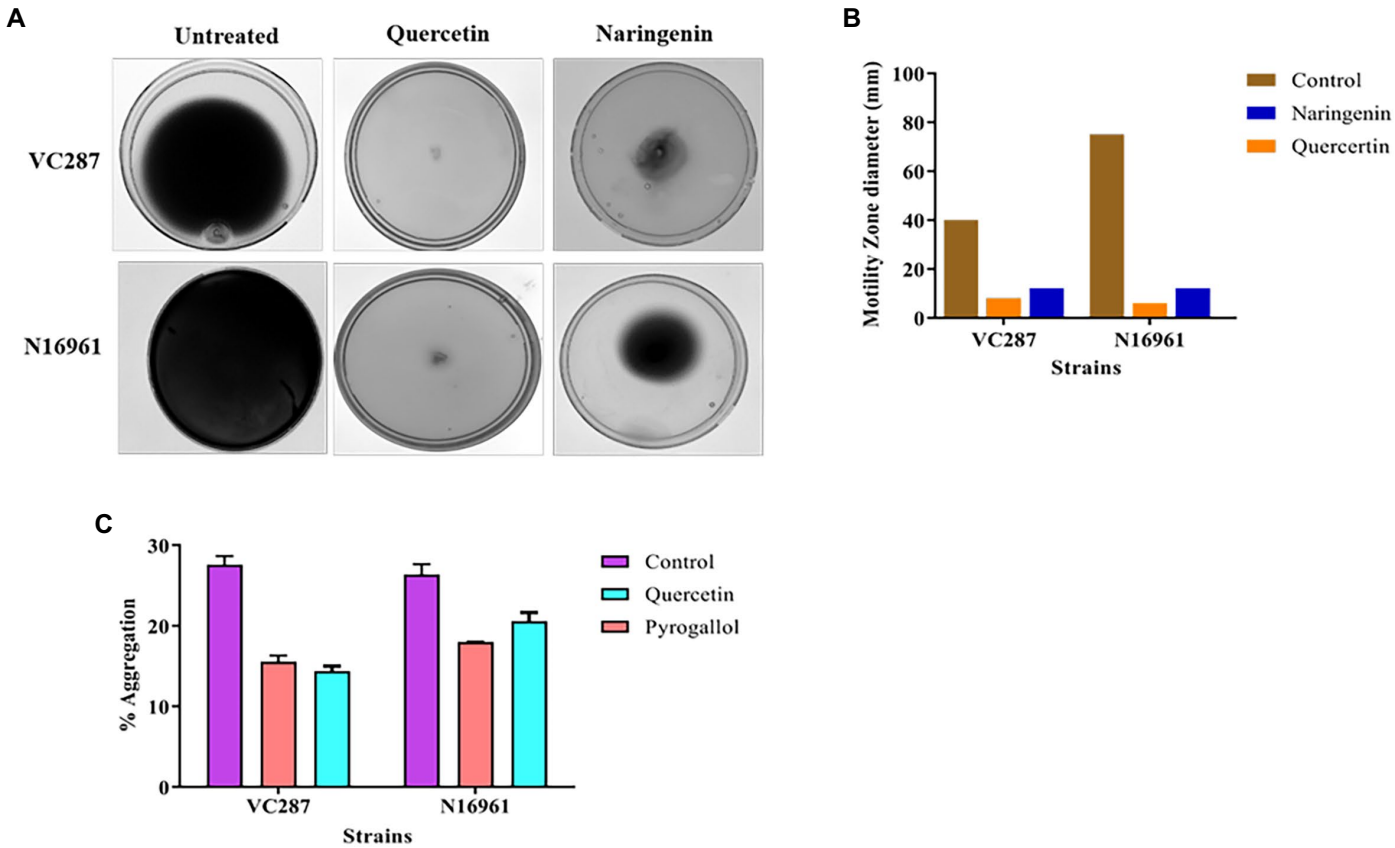
Anti-virulence assays. **(A)** The effect of quercetin and naringenin obtained on the swimming motility of *V. cholerae* strains VC287 and N16961. **(B)** Results of measurement of migration obtained with *V. cholerae* strains VC287 and N16961 in swimming motility assay. **(C)** Percentage auto-aggregation exhibited by *V. cholerae* strains VC87 and N16961 in the presence of quercetin and naringenin.

The motility of bacteria facilitates the initial attachment of *V. cholerae*. Due to the essential role of flagella in motility, the effect of quercetin and naringenin at 50 μg/ml concentration was determined in *V. cholerae* strains. Our results demonstrate that quercetin and naringenin effectively reduced the swimming motility of *V. cholerae* strains N16961 and VC287. The average diameter of the bacterial colony was significantly decreased with **≤**20 mm diameter in presence of quercetin and naringenin (Figures 8A,B).

Further, we determined the effect of quercetin, naringenin, and pyrogallol at 50 μg/ml concentration on auto-aggregation, a property often linked with biofilm formation. The untreated cells showed 25–30% autoaggregation but treated cells with quercetin and naringenin showed 15–18% autoaggregation (Figure 8C), indicating its role in biofilm formation.

### Anti-adhesion and anti-invasion assay

**Figure 9:**
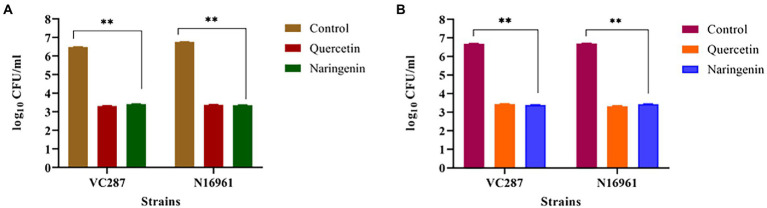
Anti-adhesion and anti-invasion effect of quercetin and naringenin obtained with *V. cholerae* strain VC287 and N16961 onto HeLa monolayer cells. **(A)** Adhesion data showed a significant reduction in the number of adhered *V. cholerae* onto the HeLa cells after treatment with quercetin and naringenin. **(B)** Invasion results show a significant reduction in number of invaded cells after treating the bacteria with quercetin and naringenin. **, *** denotes level of significance.

We examined the effect of quercetin and naringenin on the adhesion of *V. cholerae* strains to the HeLa cell monolayer. In VC287, quercetin and naringenin showed a significant reduction in the number of cells up to 2.1 × 10^4^ CFU/ml, and 1.8 × 10^4^ CFU/ml cells, respectively, compared to untreated cells 3.1 × 10^6^ CFU/ml. However, in case of N16961, the number of cells in quercetin-treated cells was 2.4× 10^4^ CFU/ml and for naringenin it was 2.1× 10^4^ CFU/ml cells compared to untreated cells 5.7 × 10^6^ CFU/ml (Figure 9A). These observations indicate that phytomolecules had anti-virulence properties.

Next, we determined the effect of quercetin and naringenin on invasion by *V. cholerae* strains with an inoculum of 10^7^ CFU/ml. In VC287, the average number of recovered bacteria was 2 × 10^4^ CFU/ml and 2.2 × 10^4^ CFU/ml after treatment with quercetin and naringenin, respectively, compared to untreated cells which were 4. 9 × 10^6^ CFU/ml. However, in N16961, the number of quercetin-treated cells was 2.2 × 10^4^ CFU/ml and of naringenin was 2.8× 10^4^ CFU/ml in comparison to untreated cells, i.e., 5.1 × 10^6^ CFU/ml (Figure 9B). This observation indicates that the inhibitors could weaken the invasive properties of the *V. cholerae* strains.

### Determining cell viability in the presence of inhibitors

**Figure 10:**
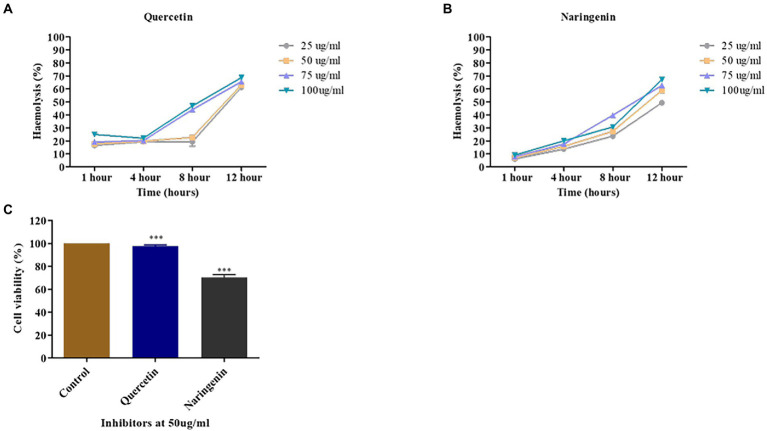
Toxicity assays. **(A)** The percent hemolysis of human red blood cells at 25, 50, 75, and 100 μg/ml concentrations of quercetin. **(B)** The percent hemolysis of human red blood cells obtained at 25, 50, 75, and 100 μg/ml concentrations of naringenin. **(C)** The viability of HeLa cells observed after exposure to quercetin and naringenin at 50 μg/ml for 24 h. **, *** denotes level of significance.

A human erythrocyte hemolysis assay was performed to see the effect of inhibitors on hemolysis at 25, 50, 75, and 100 μg/ml concentration for 1, 4, 8, and 12 h. Quercetin showed no observable hemolysis at 50 μg/ml for 8 h, but naringenin showed 25% hemolysis (Figures 10A,B). Both phytomolecules showed 70% hemolysis after an extended period of incubation (Figures 9A,B) compared to the positive control.

MTT assay was used to determine the toxic effect of quercetin and naringenin at 50 μg/ml concentration on the HeLa cell line. The results of this study showed that about 95% of HeLa cells were viable in the presence of quercetin but 70% of cells were in the presence of naringenin (Figure 10C). This finding is indicative of the fact that quercetin and naringenin are less toxic or non-toxic to cells.

### Comparative analysis of inhibitors

Violacein inhibition is one of the major criteria for comparison of the inhibitors to the known inhibitors Furanone C-30 and terrein that has anti-quorum sensing agents in several micro-organisms (He et al., 2012; García-Contreras et al., 2013; Kim et al., 2018). As compared to Furanone C30 and terrein, the candidate phytomolecules quercetin, and naringenin showed 70–85% of inhibition in violacein production and biofilm formation (Supplementary Figure S3). This finding was further confirmed by the relative expression of the quorum-sensing associated genes. There was a two-fold increase in the expression of *cqs*A and *hapR* genes but a two-fold decrease was found in the expression of *luxO* and *aphA* genes in the case of treated cells. The terrein and furanone C-30 treated cells had gene expression profiles similar to the untreated cells which further validate our initial findings.

## Discussion

Bacterial infections are becoming cumbersome day by day and the emergence of antimicrobial resistance makes bacterial infection a major threat to public health and the economy. Over the years studies demonstrated that plant extracts possessed anti-biofilm and anti-quorum sensing properties (Pederson et al., 2018). Therefore, we aimed to use broad-spectrum QS modulator molecules that are known to have anti-biofilm or anti-Quorum sensing effects in different micro-organisms.

In this study, we found that the *V. cholerae* O1 strains VC287 and N16961 formed a thicker biofilm. It has been reported that *V. cholerae* non-O1 and non-O139 strains formed moderate to high biofilm (Dua et al., 2018). We examined the efficacy of the phytomolecules for their anti-biofilm properties and found that quercetin and naringenin showed 70–80% dispersal of the biofilm. This finding is similar to those who demonstrated a significant reduction in biofilm formation on treatment with quercetin in *Pseudomonas aeruginosa* (Ouyang et al., 2016). Vikram et al. (2010) showed that naringenin lowers the amount of AHLs in *Yersinia enterocolitica*. The quorum quenching ability of secondary plant metabolites such as quercetin and naringenin was shown against *E. coli* O157:H7 (Kalia, 2013). Based on the above findings, we investigated the effect of quercetin and naringenin on the biofilm-forming strains VC287 and N16961. The data from CLSM analysis and SEM suggest that quercetin and naringenin showed a reduction in biofilm formation. Similarly, resveratrol induces reduction in *V. cholerae* biofilm formation has been reported (Augustine et al., 2014).

The phytomolecules quercetin and naringenin showed significant anti-biofilm activity in a dose-dependent manner. The MBIC of inhibitors was found to be 100 μg/ml. However, at MIC, both inhibitors showed an 80–100% reduction in biofilm. On the other hand, a sub-inhibitory concentration (50 μg/ml) of quercetin and naringenin showed a 60–70% reduction in the biofilm formation. The time-dependent biofilm inhibition study of the strains in presence of 50 μg/ml of the phytomolecules showed no significant difference in the initial stages but showed a 30–40% reduction in the intermediate stage and a 50% reduction in the maturity phase. Similar studies were conducted using naringenin against *Staphylococcus aureus* suggesting that quercetin and naringenin can work on preformed biofilms (Wen et al., 2021).

In this study, we found that quercetin and naringenin reduced 75–80% of violacein production. Similarly, a reduction in violacein production by quercetin (Bali et al., 2019) and by naringenin (Vandeputte et al., 2011) in *C. violaceum* CV026 has been reported. Further, the phytomolecules caused a bacteriostatic effect with no significant reduction in the cell growth which further emphasized the anti-quorum sensing effects of quercetin and naringenin. Similar results were reported in *Pseudomonas aeruginosa* in which 15.6 and 3.9 μg/ml of quercetin did not kill the cells rather affected the quorum sensing pathway (Taghavi et al., 2021). These observations are thus indicative of the fact that these phytomolecules can act as inhibitors against quorum sensing in *V. cholerae*. A study against *Pseudomonas aeruginosa* indicated that quercetin did not inhibit bacterial growth but led to the inhibition of biofilm formation (50%) and the production of various virulence factors like elastase, protease and pyocyanin. Quercetin at 16 μg ml^−1^ led to significant downregulation of essential QS genes including *lasI*, *lasR*, *rhlI,* and *rhlR* by 34, 68, 57, and 50%, respectively (Ouyang et al., 2016).

The metabolic activity in biofilm confirmed that the inhibitors can reduce biofilm formation without killing bacteria. This finding was substantiated by live dead staining followed by CLSM analysis that showed the reduction in the biofilm formation but without affecting the bacterial growth in the biofilm. These results are corroborated by the findings of Augustine et al. (2014), who reported that resveratrol reduced biofilm formation in *V. cholerae* without killing bacteria. The non-killing nature of the phytomolecules was further confirmed by propidium iodide staining followed by FACS analysis. Kannan et al. (2017) also reported that *V. cholerae* cells produced biosurfactants and showed 90% cell viability, indicating their non-killing mode of action. Gene expression study in *V. cholerae* strains showed two-fold downregulation of biofilm-associated genes *gbp*A, *vps*A, *rbm*A, *mba*A, and *rbm*A. Similarly, the *vps*A gene which is crucial for vibrio polysaccharide production decreased two-fold, a finding reported by Pederson et al. (2018) using cranberry extract that affected the biofilm-associated genes, and maturation of biofilm. The expression of *cqs*A and *cqsS* genes were upregulated, however, the expression of *hapR* was upregulated in the presence of phytomolecules indicating its role in decreasing biofilm formation. In addition, *hapA* gene expression was upregulated but master regulator *luxO* was downregulated causing the detachment of proteases that allow *V. cholerae* to separate itself from the small intestine and exit from the host. These findings are similar to those of Narendrakumar et al. (2019) who showed twofold downregulation in *luxO* gene expression in tryptanthrin-treated *V. cholerae*. On the other hand, *aphA* gene expression was significantly downregulated that positively regulates biofilm formation and virulence-associated genes in *V. cholerae*. This observation led to the hypothesis that phytomolecules are increasing the accumulation of the CAI-1 autoinducer, inducing a phosphorelay cascade thus decreasing the virulence factor and biofilm formation (Lu et al., 2015). However, both the phytomolecules had broad-spectrum activities so further investigations are required for finding an exact mode of action for these phytomolecules. In a recent study against wild-type strains of *V. harveyi,* the crude extract of *P. betle* was able to inhibit biofilm formation and bioluminescence. The extract was found to affect several QS genes, *luxM*, *luxN*, *luxP*, *luxQ*, *luxS*, *luxO*, and *luxR*. These results led to their conclusion that *P. betle* crude extract is probably targeting the AI-1 and AI-2 QS pathways of *V. harveyi*, thereby affecting its virulence (Guzman et al., 2022).

In another study a new strain, assigned as XTS1.2.9, a *Vibrio parahaemolyticus*, the cell free supernatant showed inhibitory effect on quorum sensing in *Vibrio harveyi* by targeting its quorum-sensing sensor 1, especially sensor CAI-1 (Hoang et al., 2022).

Aggregation is one of the essential factors for the initiation of *V. cholerae* biofilm formation and it is an important criterion for verifying the anti-biofilm ability of inhibitor molecules. Bhattacharya et al. (2020) demonstrated that pentacyclic triterpenoids affected auto-aggregation in *V. cholerae* strains. In this study, we found that quercetin and naringenin reduced auto-aggregation compared to the untreated cells, thus indicating that phytomolecules had the potential to interfere at various stages of biofilm maturation. Also, we found that 50 μg/ml concentration of quercetin and naringenin inhibited bacterial motility, thus confirming the efficacy of phytomolecules against *V. cholerae* virulence.

We assessed the abilities of quercetin and naringenin to inhibit adherence and invasion capability of *V. cholerae* strains to the epithelial cell line HeLa. Quercetin and naringenin showed a reduction of bacterial cells and were substantiated by downregulation in *tcpA* gene expression. Hema et al. (2017) demonstrated the anti-adhesion effect of PDCA^py^ with INT407 cells that showed a significant reduction of 1.5 × 10^5^ CFU/ml and an anti-invasion effect showing a higher reduction of 2.2 × 10^4^ CFU/ml in *V. cholerae*. These observations thus suggest that *tcpA* expression can be correlated with adhesion and invasion properties of *V. cholerae*.

Terrain and furanone C-30 are used as a positive control against many gram-negative bacteria to find alternative or new quorum-sensing inhibitors (Aburto-Rodríguez et al., 2021). Therefore, we compared the inhibition of quercetin and naringenin with terrein and furanone C-30 and found that quercetin and naringenin showed a significant reduction in violacein production compared to terrein and furanone C-30. Also, we observed that *hapR* expression was upregulated by quercetin and naringenin thus regulating the function of the *luxO* gene. However, there was no change in the expression profile of these genes, biofilm formation, and violacein production in presence of terrein and furanone C30. These finding thus suggests that terrein and Furanone C-30 had species-specific activity but quercetin and naringenin can be used as broad-spectrum quorum sensing modulators but need further study.

The toxicity of molecules is one of the important factors when considering a candidate for combination therapy. The phytochemicals used in this study showed better viability of the HeLa cells and less hemolysis of H-RBC thus suggesting their non-toxic nature. These results corroborate with the finding of Narendrakumar et al. (2019) who also found 90% cell viability in HEK cells and less than 10% hemolysis with tryptanthrin used against *V. cholerae*. This finding thus suggests that quercetin and naringenin are safe and can be considered candidates for quorum sensing modulators.

## Conclusion

We attempted to understand the effect of phytomolecules on the biofilm formation of the *V. cholerae* strains. Based on the results obtained in this study, we hypothesize that quercetin and naringenin affect biofilm architecture, virulence factor, and biofilm formation. Thus, we propose that the phytomolecules quercetin and naringenin can act as promising anti-biofilm and anti-quorum sensing agents. However, an in-depth investigation of the mode of action of these phytomolecules will help us to find better therapeutics against *V. cholerae* infections.

## Data availability statement

The original contributions presented in the study are included in the article/Supplementary material, further inquiries can be directed to the corresponding author.

## Ethics statement

This study uses strains obtained from Clinical and Environmental Sources, and the Institutional Human Ethics Committee of the Institute of Life Sciences did not require the study to be reviewed or approved because the strains were from laboratory stock. Written informed consent for participation was not required for this study in accordance with the national legislation and the institutional requirements. The data were analyzed anonymously and reported.

## Author contributions

SS and DS conceived and designed the experiments. SS and SA experimented the study. SS and DS analyzed the results. SS, SA, and DS wrote the manuscript. All authors reviewed and approved the manuscript.

## Funding

In part, the study was supported by funds from the Department of Biotechnology, New Delhi, to the Institute of Life Sciences, Bhubaneswar. SS is grateful to the Department of Science and Technology for providing INSPIRE Senior Research Fellowships and SA to the Institute of Life Sciences, Bhubaneswar, for providing Senior Research Fellowships. The funder had no role in the study design, data collection, analysis, or decision to publish or prepare the manuscript.

## Conflict of interest

The authors declare that the research was conducted in the absence of any commercial or financial relationships that could be construed as a potential conflict of interest.

## Publisher’s note

All claims expressed in this article are solely those of the authors and do not necessarily represent those of their affiliated organizations, or those of the publisher, the editors and the reviewers. Any product that may be evaluated in this article, or claim that may be made by its manufacturer, is not guaranteed or endorsed by the publisher.

## Footnotes

^1^ https://www.who.int/news-room/fact-sheets/detail/cholera
