# Supplementary material for: Attenuation of quorum sensing system and virulence in Vibrio cholerae by phytomolecules
Source: Front Microbiol. 2023 Mar 30;14:1133569. doi: 10.3389/fmicb.2023.1133569 (PMC10098448; doi:10.3389/fmicb.2023.1133569)
Supplement: Supplementary file 1 [file Data_Sheet_1.pdf]

Supplementary materials

Materials and Methods

Dose-dependent effect of inhibitors on biofilm

We determined the inhibitory effect of quercetin and naringenin on biofilm of *V. cholerae* strains N16961 and VC287. The phytomolecules were added at concentrations 25µg/ml, 50µg/ml, 75µg/ml, 100µg/ml, and 125µg/ml to determine their effect on the biofilm. The plates were incubated for a total of 24 hrs at 37°C under static condition and the wells were treated with inhibitors and allowed to incubate for next 24 hrs.

Time Point Inhibition Assay

We added quercetin, naringenin and pyrogallol to the wells containing *V. cholerae* strains N16961 and VC287 at 0, 8, 12, 16, 20 and 24 hrs to see the effect on biofilm formation at sub-MBIC concentration determined from the above-mentioned experiments. All wells were incubated for a total of 24 hrs at 37°C under static condition and were further incubated for another 24 hrs after the addition of the inhibitors.

Comparative analysis of inhibitors

The effect of the inhibitors was tested in the *V. cholerae* strains in comparison to terrein and Furanone C-30 which are well known quorum sensing blockers for several bacterial species. The comparative inhibitory effects were tested against *C. violaceum* CV026. The violacein pigment were extracted and quantitatively analyzed. The inhibitors were then tested against *V. cholerae* biofilms and q-RT PCR was performed to determine the effect of inhibitors on the QS associated genes (Rodríguez et al., 2021).

Results

Dose dependent inhibition of biofilm formation

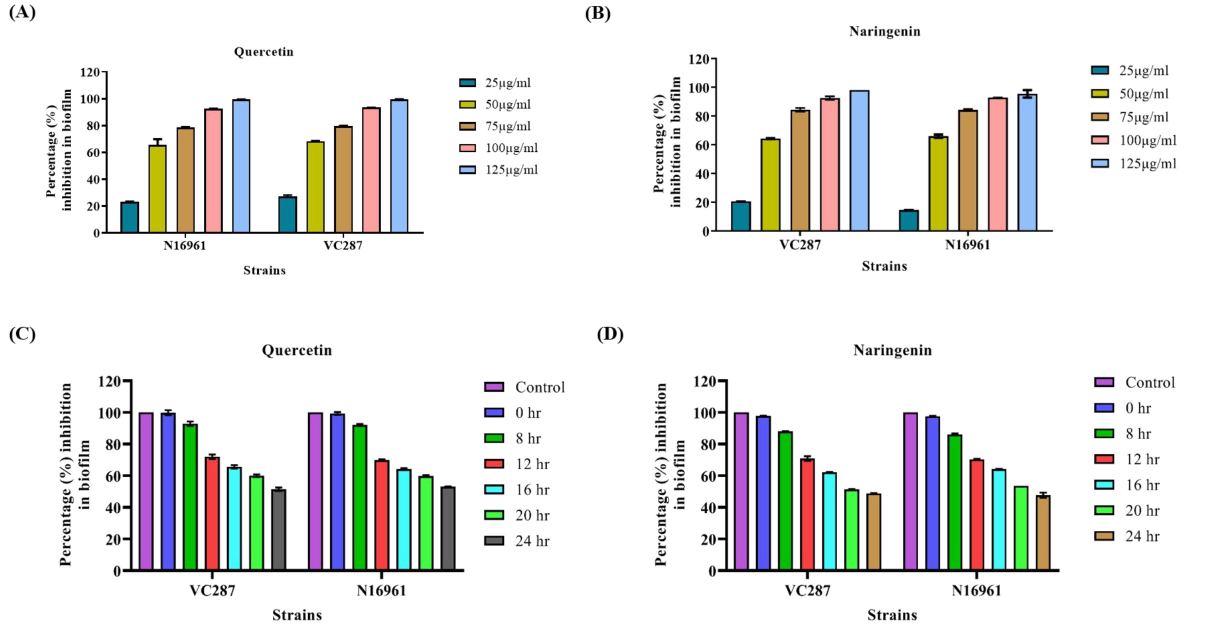

Supplementary Figure 1. Effect of different concentrations of quercetin and naringenin on biofilm formation (expressed as percentage of inhibition) on *V. cholerae* strains (A) N16961 and (B) VC287. The effect of quercetin and naringenin on biofilm formation by *V. cholerae* strains at different time points (C) N16961 and (D) VC287.

CLSM analysis biofilm formed by *V. cholerae* strains

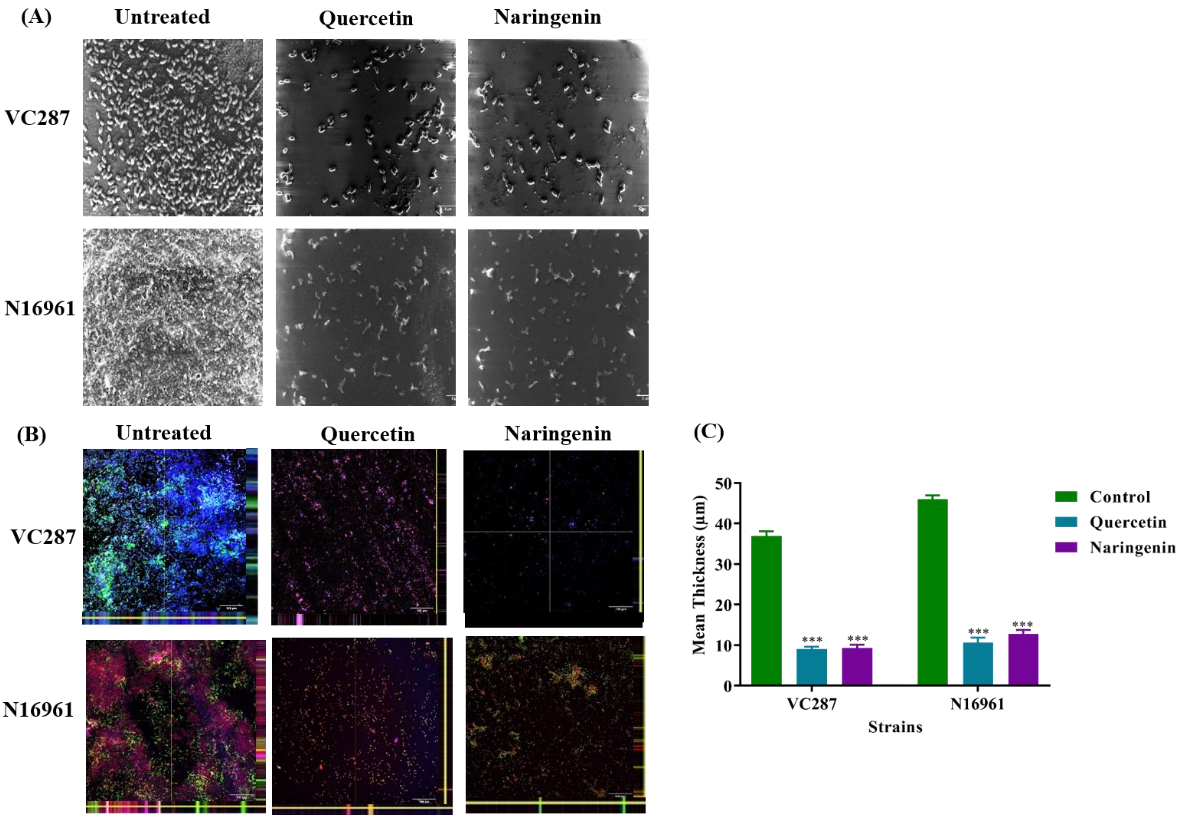

Supplementary Figure 2. Scanning electron microscopy of *V. cholerae* strains N16961and VC287 in the absence and presence of quercetin and naringenin. Orthogonal views obtained by confocal laser scanning microscopy of 24-hr grown culture of N16961 and VC287. The z-stacks were acquired for each chamber with Leica TCS SP5 confocal scanning system (Leica Microsystem, Mannheim, Germany) using 63× oil objective lens. CLSM analysis represented as mean thickness of biofilm formed by *V. cholerae* strains.

Comparative analysis of inhibitors

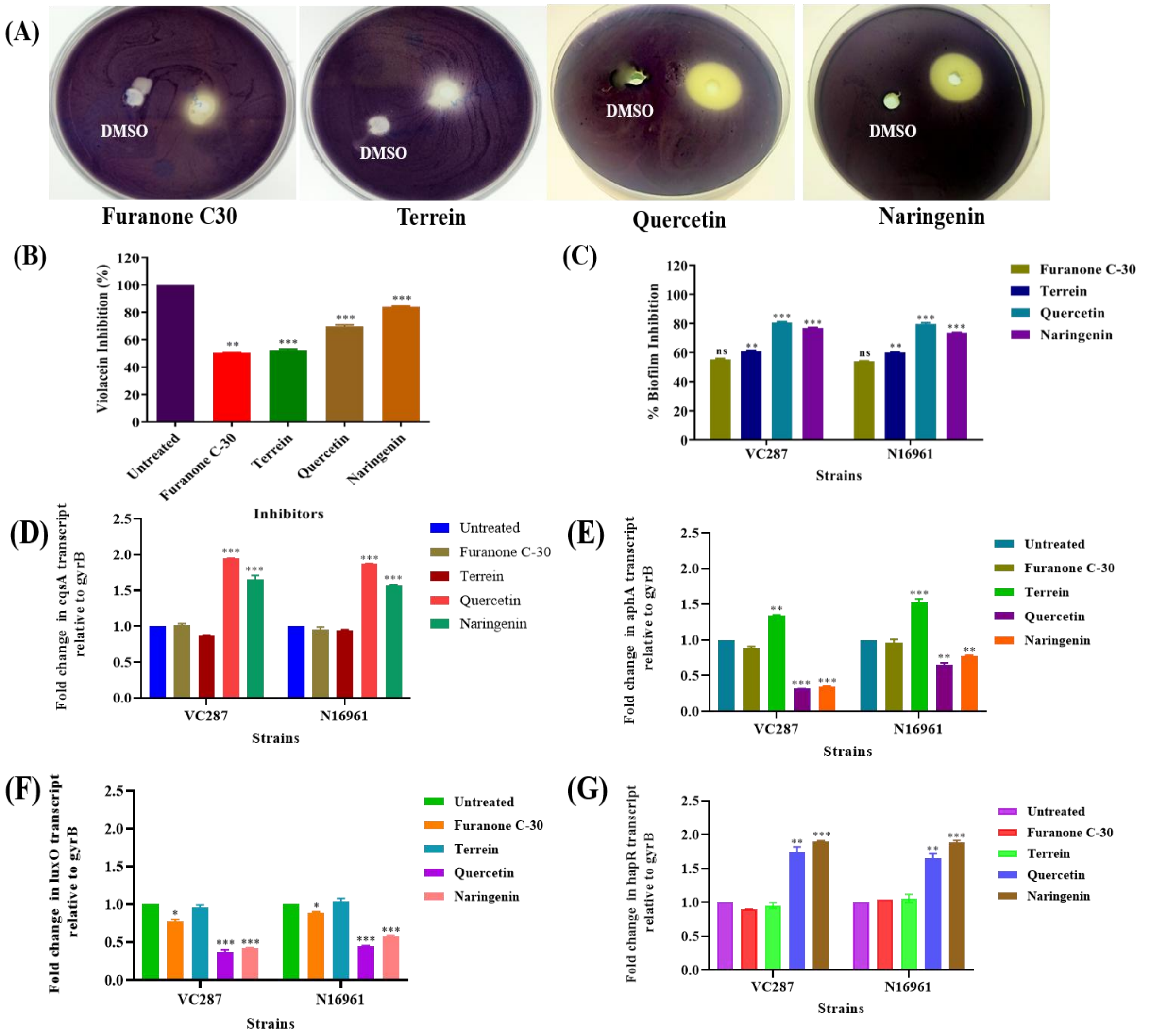

Supplementary Figure 3. Quorum sensing of *C. violaceum* CV026 by quercetin and naringenin (A) The zone of inhibition at 50 µg/ml concentration of inhibitors with DMSO as control. (B) Quantitative estimation of violacein production (%) with and without the inhibitors. (C) Percentage of biofilm inhibition in the presence and absence of inhibitors (D) Relative expression of quorum-sensing genes measured by real-time qPCR. (D) *cqs*A; (E) *aph*A; (F) *lux*O; (G) *hap*R; The *V. cholerae* strains VC287 and N16961 cells were tested in the absence (control) and presence of inhibitors; pyrogallol, quercetin, naringenin and commercially available QS-blocker Furanone C-30 and terrein for 24 hrs. The fold changes of QS gene expression were normalized to the level of *gyr*B gene expression and then quantitated relative to gene expression of control was normalized to 1 using the comparative Ct method. Results represent the means and SD from triplicate experiments. *P < 0.05 and **P < 0.01 indicate statistically significant differences between treated and control.

References

1. Aburto-Rodríguez, N. A., Muñoz-Cázares, N., Castro-Torres, V. A., González-Pedrajo, B., Díaz-Guerrero, M., García-Contreras, R., ... & Martínez-Vázquez, M. (2021). Anti-pathogenic properties of the combination of a T3SS inhibitory halogenated pyrrolidone with C-30 furanone. *Molecules*, 26(24), 7635.
2. García-Contreras, R., Martínez-Vázquez, M., Velázquez Guadarrama, N., Villegas Pañeda, A. G., Hashimoto, T., Maeda, T., ... & Wood, T. K. (2013**).** Resistance to the quorum-quenching compounds brominated furanone C-30 and 5-fluorouracil in *Pseudomonas aeruginosa* clinical isolates. *Pathogens and disease,* 68(1), 8-11.

3. He, Z., Wang, Q., Hu, Y., Liang, J., Jiang, Y., Ma, R., ... & Huang, Z. (2012). Use of the quorum sensing inhibitor furanone C-30 to interfere with biofilm formation by *Streptococcus mutans* and its *luxS* mutant strain. *International journal of antimicrobial agents,* 40(1), 30-35.

4. Kim, B., Park, J. S., Choi, H. Y., Yoon, S. S., & Kim, W. G. (2018). Terrein is an inhibitor of quorum sensing and c-di-GMP in *Pseudomonas aeruginosa*: a connection between quorum sensing and c-di-GMP. *Scientific reports,* 8(1), 1-13.
